# 20-m resolution longitudinal nitrate data from before and after rainfall in low-order streams spanning rural to urban gradients

**DOI:** 10.1016/j.dib.2021.107256

**Published:** 2021-07-20

**Authors:** Colleen Bradley, P. Zion Klos

**Affiliations:** a Undergraduate Research Program, School of Science, Marist College, Poughkeepsie, New York, USA; b Department of Environmental Science and Policy, Marist College, Poughkeepsie, New York, USA

**Keywords:** Water quality, Nutrient loading, Landscape run-off, Intensified precipitation, Nonpoint source pollution, Hudson River Valley, Northeast US, Fall Kill, Sprout Creek

## Abstract

This study aimed to provide datasets useful for identifying and disentangling any complex patterns in the fine spatial heterogeneity of a common nonpoint source pollutant, nitrate, as well as other related water quality metrics (temperature, dissolved oxygen, pH, and conductivity) in streams of the eastern U.S. We aimed specifically to provide a resource dataset of uniquely high spatial detail for the better understanding of how the rapid appearance or disappearance of this common nutrient at fine scales may relate to other aspects of form and function in these watersheds. To accomplish this, the low-order streams observed in this study extend across environmental gradients in urban and rural land cover, and change from free flowing to impounded water. Study streams were also observed at different levels of steam discharge, both pre and post summer storm events. Four unique 600 m stretches of two low-order creeks in Dutchess County, New York, were observed longitudinally at a fine spatial resolution (20 m). These four 600 m stretches contrasted in land cover from heavily urban channelized streams to mainly rural meandering streams with seasonally active floodplains. Data collection occurred above and below areas where impounded water was input into to the stream via a side tributary to observe the effect of adjacent impoundments, and fine scale water quality was assessed both before and after major storm events to see the influence of wet-period flow path activation on nonpoint source fluxes. Data was organized into a final data table and maps showing how water quality parameters change in regard to land use and land cover, free flowing versus impounded water, and the influence of storm events versus dry periods. Water quality data are also mapped in comparison to thresholds of concern for growth of cyanobacteria – a common constituent of harmful algal blooms in the region. Fine spatial resolution data from this study can be used as a baseline for future studies or modeling efforts at similarly fine scales to better understand why concentrations of nitrate rapidly rise or fall longitudinally at <100 m scales within these forms of streams, and how land cover change and intensified storms due to climate change may further influence water quality dynamics at these uniquely fine scales.

## Specifications Table

**Table:** 

Subject	Earth-Surface Processes
Specific subject area	Hydrology of non-point source pollution at a fine spatial scale
Type of data	Figures Tables
How data was acquired	Field-based longitudinal mapping of in-stream sites (thalweg) every 20 m along four 600 m stream sections. At each site, depth (cm), width (m), pH, temperature (°C), nitrate (mg/L), dissolved oxygen (mg/L), and conductivity (µS/cm) were collected. Instruments: YSI model 6,050,000: Professional Plus Multiparameter Instrument YSI model 605,101: pH sensor YSI model 006,560: Temperature/ Conductivity sensor YSI model 605,106: Nitrate ISE sensor YSI model 605,203: Polarographic dissolved oxygen sensor Google LLC Version 5.43: Google Maps Westcott product number ACM0432: Meter stick wood ruler Crescent Lufkin model FE300: 300 ft tape measure
Data format	Raw Analyzed
Parameters for data collection	Two streams of similar order with a water impoundment that connects to each stream by a tributary that separates the upper and lower 600 m sections. The streams exist with different land use / land cover, with one predominantly urban versus one predominantly rural, which creates contrasting forms of stream morphology with urban being heavily channelized and rural mainly meandering. Also, both streams are in close spatial proximity (adjacent watersheds) so as to have approximately the same weather events and underlying geology.
Description of data collection	A minimum of 30 stream sampling sites were manually mapped out 20 m apart. At each site depth (cm, thalweg) and stream width (m) were measured. A YSI Multiparameter Quad-Probe was calibrated before each field campaign, and then submerged at each site to measure pH, temperature (°C), nitrate (mg/L), dissolved oxygen (mg/L), and conductivity (µS/cm) *in-situ*. This process was conducted at 600 m sections in two streams above and below a water impoundment and pre and post summer storm events in 2019.
Data source location	Institution: Marist College City/Town/Region: Poughkeepsie, Hudson River Valley, New York Country: United States Latitude and longitude for collected samples/data: 41.7208331, −73.9320898
Data accessibility	All data is included with the article as a .csv file.

## Value of the Data

• This data provides the unique fine spatial resolution changes of water quality and nutrients, showing how they rapidly rise and fall in low-order streams due to complex in-stream and hyporheic processes, which can aid in better understanding these processes.
• This data can be of benefit locally and globally by future researchers to use a baseline or as a comparison to other studies that investigate longitudinal nutrient dynamics in stream systems, particularly rapid increases and decreases at sub-kilometer scales.
• This data can be used to understand how intensified precipitation under climate change may change nutrient loading and remediation in urban versus rural streams [1], and better predict potential downstream issues involving eutrophication of aquatic systems, such as harmful algal blooms [2].
• This data can be used to validate or improve models investigating the fine-scale impacts that longitudinal changes in land use and land cover type, as well as differences in fluvial geomorphology and stream form, may have on common water quality metrics observed at this uniquely high 20-m resolution.
• This data is limited to only the days and sites investigated, and the values presented could be highly variable through time due to changes locally or upstream in each of these low-order watersheds; certain metrics were found to rise and fall rapidly longitudinally, so the resulting downstream inputs to the Hudson River are unknown.

## 1 Data Description

**Fig. 1:**
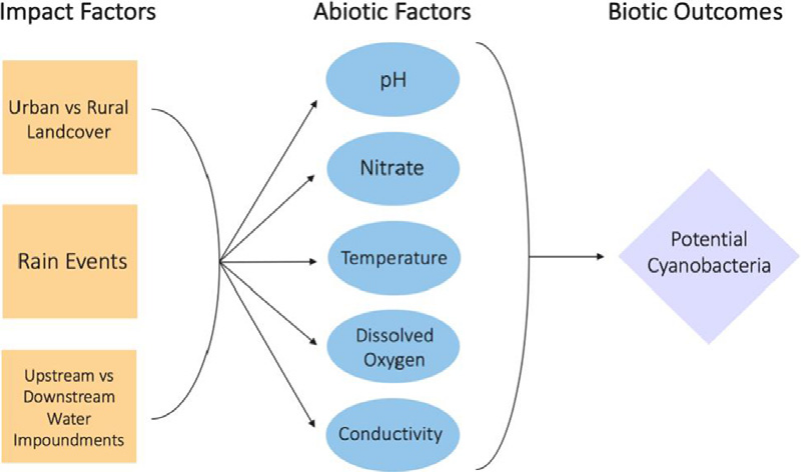
The study design.

**Fig. 2:**
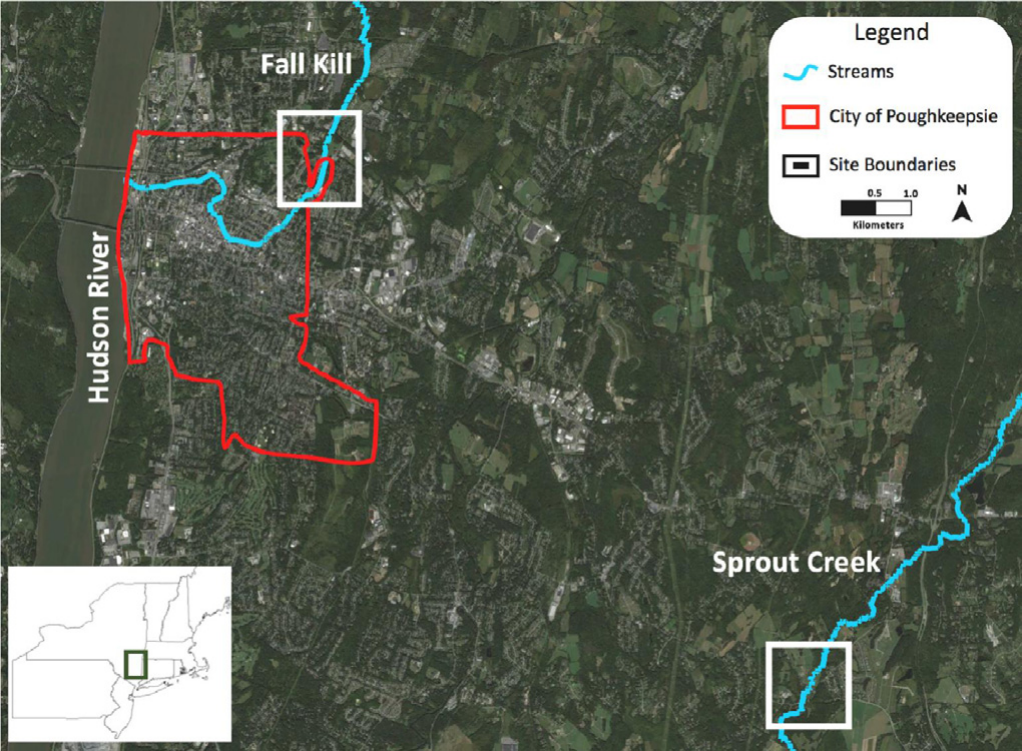
Site selections near Poughkeepsie in Dutchess County, New York.

**Fig. 3:**
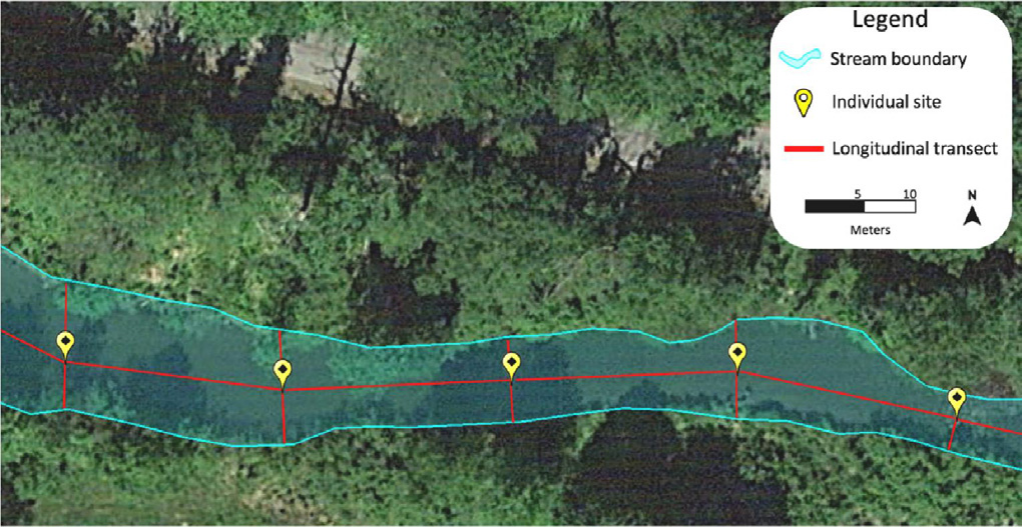
The methodology process for fine-sale data collection.

The data presented were collected through a study designed to be useful in analyzing abiotic water quality factors as they relate to surrounding land cover, storm events, and in-stream impoundments. Fig. 1 contains initial analysis of water quality measurements compared to thresholds for potential growth of cyanobacteria – the common driver of Harmful Algal Blooms (HABs) in the region. Fig. 2 shows the two streams that were chosen as the site selections near Poughkeepsie in Dutchess County, New York. The Fall Kill was the location for urban water quality measurements and Sprout Creek was the location for rural water quality measurements. Both streams are part of the greater Hudson River watershed. Fig. 3 represents the methodology used which focused on fine spatial-scale data collection. Individual sites were manually mapped out in the thalweg of the stream bed 20 m apart along a longitudinal transect. At each site the following water quality metrics were collected, pH, temperature (°C), nitrate (mg/L), dissolved oxygen (mg/L), and conductivity (µS/cm). Table 1 is raw data of the water quality metrics collected. Fig. 5, Fig. 6, Fig. 7, Fig. 8, 10 and 11 show raw data values that were collected at individual sites, and they are highlighted in bold at sites where water quality values met or exceeded water quality thresholds conducive for cyanobacteria growth in freshwater systems in the northeastern United States outlined in Table 1.

**Table 1:** Water quality threshold standards conducive to cyanobacteria growth in fresh-water systems located in the northeastern United States [3], [4], [5], [6], [7].

Abiotic factor	Water quality standards
pH	≥8.0
Nitrate (mg/L)	≥10.0
Temperature (℃)	≥20.0
Dissolved Oxygen (mg/L)	≤3.0
Conductivity (µS/cm)	≥1000.0

**Fig. 4:**
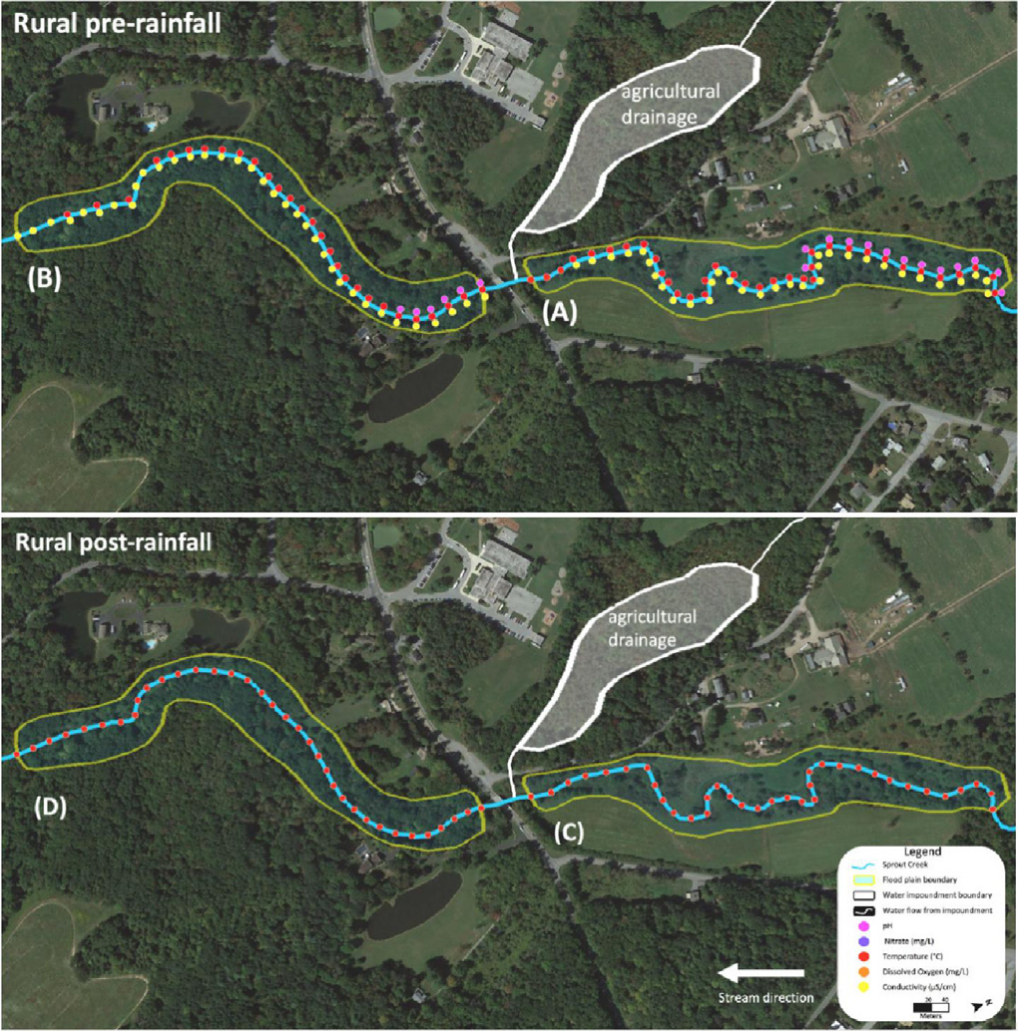
Comparison of Sprout Creek water quality data upstream and downstream a water impoundment before and after a rain event.

**Fig. 5:**
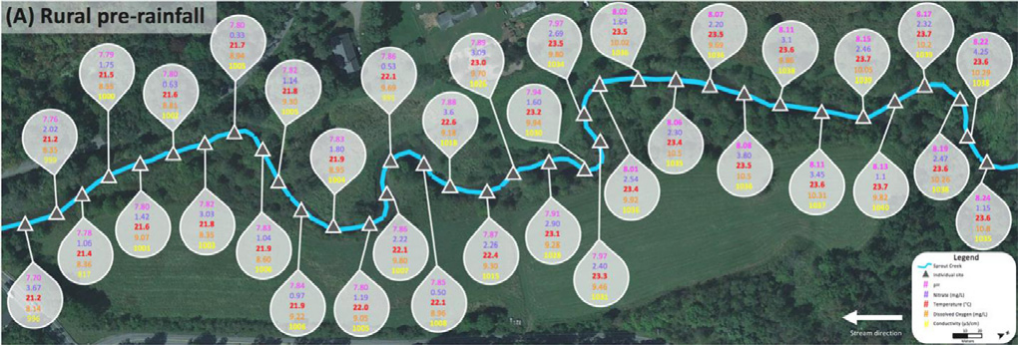
Section (A) of Sprout Creek (upstream of the water impoundment) before a rain event.

**Fig. 6:**
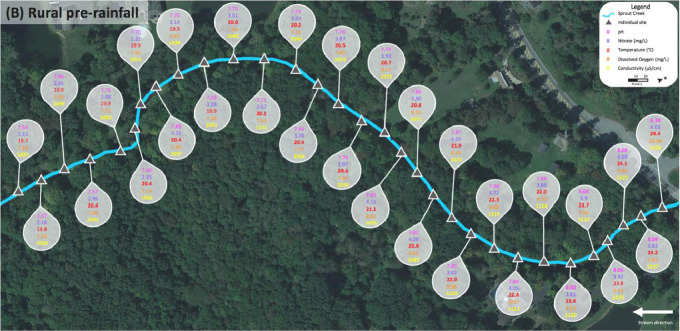
Section (B) of Sprout Creek (downstream of the water impoundment) before a rain event.

**Fig. 7:**
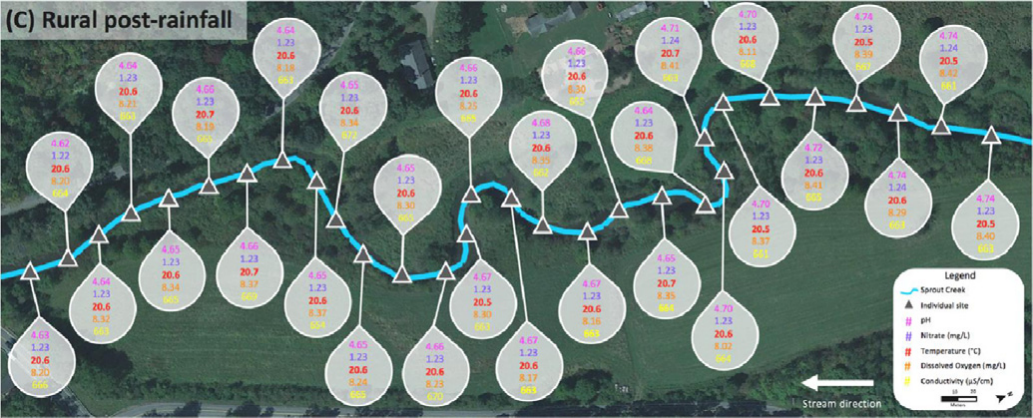
Section (C) of Sprout Creek (upstream of the water impoundment) after a rain event.

**Fig. 8:**
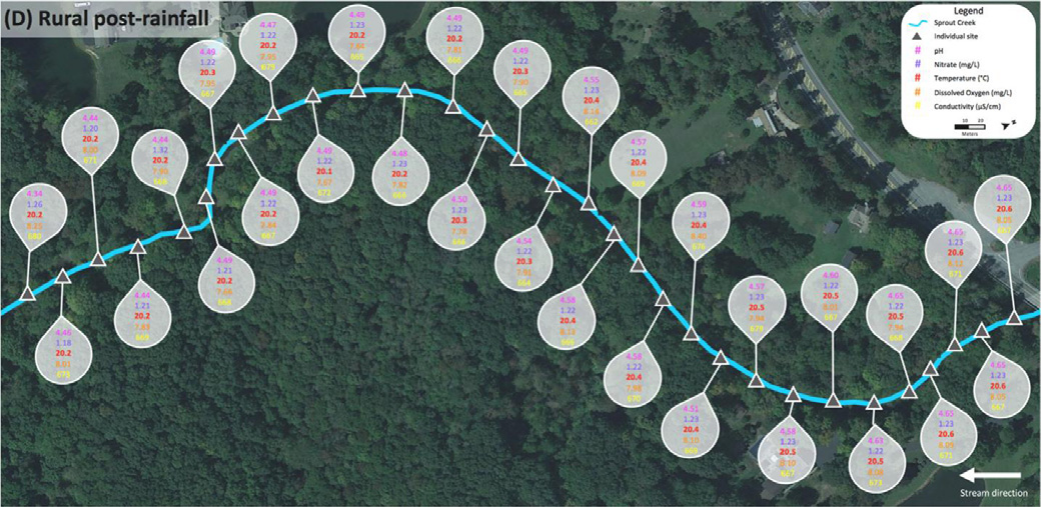
Section (D) of Sprout Creek (upstream of the water impoundment) after a rain event.

Data for the rural Sprout Creek was collected in two sections, upstream and downstream of a water impoundment, which was dominated by pastoral agricultural drainage. Fig. 4 shows sites where water quality abiotic factors were measured above the favorable standards for cyanobacterial growth in Table 1, and are dotted in corresponding colors before and after a rain event. In Fig. 4, upstream of the water impoundment pre-rainfall is section (A) and downstream of the water impoundment pre-rainfall is section (B). Upstream of the water impoundment post rainfall is section (C) and downstream of the water impoundment post rainfall is section (D). Fig. 5, Fig. 6, Fig. 7, Fig. 8 show the raw data values collected in rural Sprout Creek upstream and downstream of a water impoundment before and after a rain event.

**Fig. 9:**
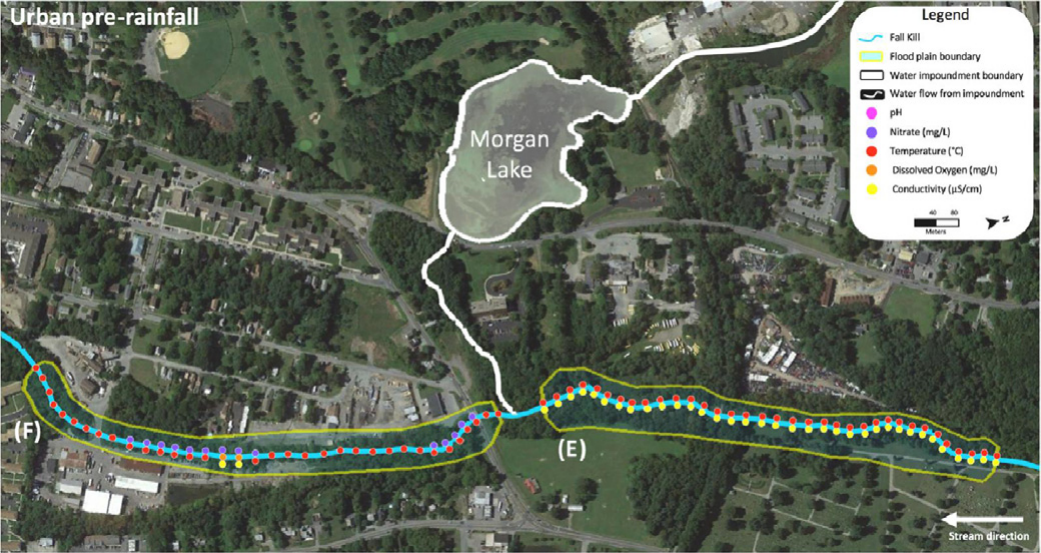
Fall Kill water quality data upstream and downstream a water impoundment before a rain event.

**Fig. 10:**
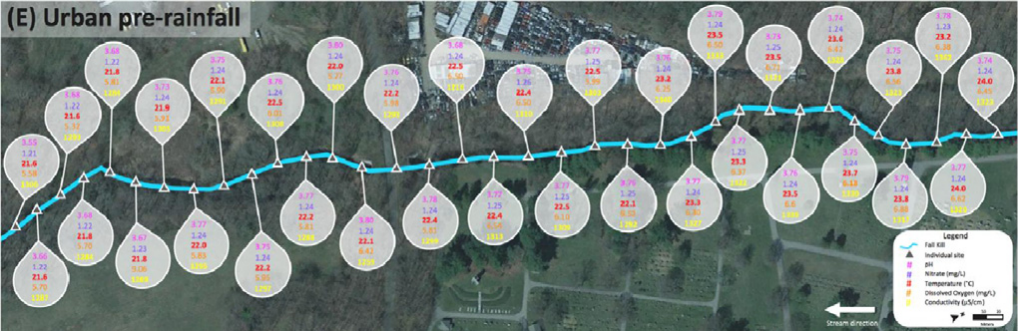
Section (E) of the Fall Kill (upstream of the water impoundment) pre-rainfall event.

**Fig. 11:**
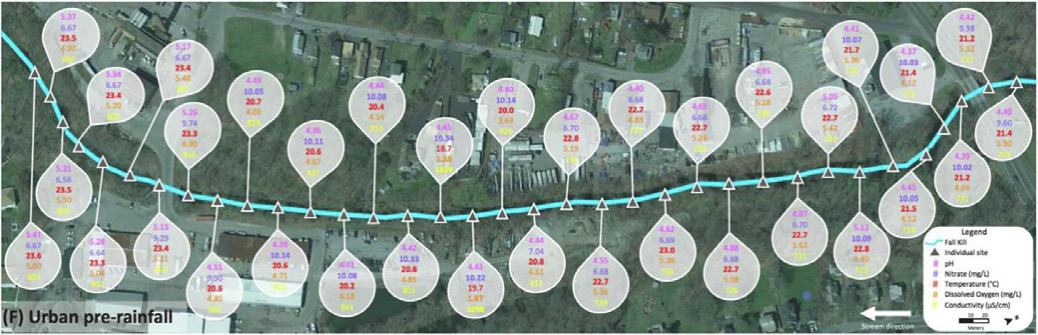
Section (F) of the Fall Kill (downstream of the water impoundment) pre-rainfall event.

Data for the urban Fall Kill was collected in two sections, upstream and downstream of a water impoundment, which was Morgan Lake adjacent to a golf course. Fig. 9 shows upstream of the water impoundment pre-rainfall in section (E) and downstream of the water impoundment pre-rainfall in section (F), and sites where water quality metrics were measured above favorable standards for cyanobacterial growth (Table 1) are dotted in corresponding colors. Figs. 10 and 11 show raw data values collected in the urban Fall Kill before a rain event; there is no post-rainfall data from the Fall Kill. All data collected at individual sites are highlighted in bold when water quality metrics met or exceeded values in Table 1.

Table 2 presents analyzed mean values from each site of the collected water quality abiotic factors. The highlighted means indicate significant changes (two-tailed Student's T-test, *p* < 0.05) related to the impact factors on the left. Table 3 provides all raw data associated with all tables and figures.

**Table 2:** Means of the collected water quality metrics. The highlighted means indicate significant changes (two-tailed Student's T-test, *p* < 0.05) related to the paired impact factors on the left.

	pH	Nitrate (mg/L)	Temperature (℃)	Dissolved Oxygen (mg/L)	Conductivity (µS/cm)
Rural	7.87	2.73	22.1	8.89	1053
Urban	4.24	4.75	22.0	5.53	1054
Before Rainfall	7.87	2.81	21.9	8.89	1054
After Rainfall	4.60	1.23	20.5	8.12	667
Rural Upstream	7.94	2.05	22.6	9.49	1015
Rural Downstream	7.80	3.45	21.6	8.42	1096
Urban Upstream	3.75	1.24	22.7	6.26	1306
Urban Downstream	4.73	8.26	21.4	4.80	799

## 2 Experimental Design, Materials and Methods

Each site was manually measured 20 m apart by a Crescent Lufkin 300 ft tape measure. Each site's latitude and longitude were recorded in Google Maps using an iPhone GPS. Depth (cm) was measured with a Westcott meter stick wood ruler and width (m) was measured by a Crescent Lufkin 300 ft tape measure. A YSI Professional Plus Multiparameter was submerged and collected (pH), temperature (°C), nitrate (mg/L), dissolved oxygen (mg/L), and conductivity (µS/cm) *in-situ* (probe specifications listed in the Specification Table above). The methods were conducted for an area of 600 m (in 20 m sections) both upstream and downstream of each water impoundment inflow tributary. The methods were repeated during dry periods at Sprout Creek on July 11, 15, and at Fall Kill on July 16 and August 14, 2019, as well as after a storm event at Sprout Creek's sites on July 23, 2019. Data was then complied in *Microsoft Excel* and mapped on *Google Earth Pro*.

## CRediT Author Statement

**Colleen Bradley**: Conceptualization, Methodology, Software, Validation, Formal analysis, Investigation, Resources, Data curation, Writing – original draft, Visualization, Supervision; **P. Zion Klos**: Conceptualization, Methodology, Validation, Formal analysis, Investigation, Resources, Writing – review & editing, Visualization, Supervision, Project administration, Funding acquisition.

## Declaration of Competing Interest

The authors declare that they have no known competing financial interests or personal relationships which have, or could be perceived to have, influenced the work reported in this article.

## Appendix Supplementary materials

Table 3. All collected data, downloadable as a comma delimited table (Full_Dataset-Bradley_Klos.csv). Image, application 1
